# An Analysis of Anger in Adolescent Girls Who Practice the Martial Arts

**DOI:** 10.1155/2011/630604

**Published:** 2011-11-24

**Authors:** Sara Lotfian, Vahid Ziaee, Homayoun Amini, Mohammad-Ali Mansournia

**Affiliations:** ^1^Sports Medicine Research Center, Tehran University of Medical Sciences, No. 7 Al-e-Ahamd Highway, P.O. Box 14395-578, Tehran, Iran; ^2^Growth and Development Research center, Tehran University of Medical Sciences, Tehran, Iran; ^3^Department of Psychiatry, Tehran University of Medical Sciences, Tehran, Iran

## Abstract

The effect of martial arts on adolescents' behavior, especially aggression, is controversial. The aim of this study was to assess and compare anger ratings among adolescent girl athletes of different martial arts. 291 female adolescents between the ages of 11 and 19 were assessed according to the Adolescent Anger Rating Scale designed by DM Burney. In the case group, the martial arts practiced were either judo (*n* = 70) or karate (*n* = 66), while the control group was composed of swimmers (*n* = 59) and nonathletes (*n* = 96). Total anger scores showed statistically significant differences between the groups (*P* = 0.001) decreasing from girls who practiced judo to nonathletes, karate, and swimmers. Instrumental and reactive anger subscales also showed significant differences between the groups, but this difference was not found for anger control. As a conclusion, the anger rate did not differ between judoka and nonathletes, but that both of these groups received higher scores in total anger than karateka and swimmers.

## 1. Introduction

The practice of martial arts has grown significantly in recent decades. Every year official competitions of these sports are held regularly, and the number of participants is increasing. This trend has led to increased participation by both male and female adolescents in these sports. Karate and judo are among the more popular of the martial arts, especially in Eastern countries.

The word “karate” means “empty hand”; it is one of the old Japanese martial arts that has gradually become a combat sport. Karate practice can be divided into three parts: kihon, kata, and kumite. “Kihon” are basic techniques, “kata” are set combinations of techniques which are practiced with an imaginary opponent, and “kumite” is sparring with an opponent which involves the use of controlled kicks and punches [1].

The word “judo” means “the gentle way” and its origin, like karata, comes from Japan. Accordingly, its philosophy is to teach the individual to deal with problems in a gentle way and is meant to contribute not only to physical development but also to psychological maturity [2]. 

Because of the clashing nature of these kinds of sports, there have always been questions regarding their effect on adolescents' behavior. Some of these questions concern how they may affect the rate of aggression among adolescents. Questions like “can exercise lead to an increase or decrease in adolescents' aggression?”, and “which kind of sport has a negative and which has a positive effect on athletes' aggression?”, “are aggressive individuals more inclined to a special kind of sport?” [3, 4] need to be answered. Prior studies have not offered a definitive answer to these questions and the subject remains controversial, although not one study is able to answer them. 

Aggression is a forceful, goal-directed action that may be verbal or physical and is the motor counterpart of the affect of rage, anger, or hostility [5]. Although participation in sports has many positive aspects, championship tournaments usually involve aggressive behavior [6]. Aggression can be divided into two types, hostile and instrumental. The goal of hostile or reactive aggression, which is motivated by anger, is to inflict injury or psychological harm on someone else without a further goal. By contrast, in instrumental aggression the goal is to injure an opponent in order to increase one's chances of victory [6, 7]. Primary studies of aggression in athletes who practice high physical contact sports, that is, football players, judokas, or wrestlers were more aggressive, in both sports and in their daily lives, than athletes who practiced low or medium contact sports [3]. Following this theory, complementary studies were carried out in different sports, in which the results showed that in martial arts the aggressiveness levels decrease with the duration of practice in martial arts [4, 8]. Some researchers have proposed martial arts as a way of the acquisition of emotional and behavioral self-control [4, 8], while others have proposed that karate or judo training would not lead to a decrease in aggression [9–11]. The small sample size of previous studies justifies the need for more research in this regard. On the other hand, the question of whether aggressive individuals are more inclined to participate in these kinds of sports or martial arts, and the effect that such a confusion factor may have on aggressiveness levels among athlete, has increased the ambiguity of previous studies [3].

Some studies have shown no difference in the aggression scores of children beginning karate training compared to the control group, but a higher anger score in children beginning judo training has been found [3]. An absence of differences in aggression scores between children who participate in karate compared to a control group [11] and a significant increase of aggression among children studying judo [12] have also been reported. The results suggested that kata or meditation training can lead to a reduction of aggression among karate athletes [10].

There are a limited number of research studies concerning aggression and anger among young martial arts athletes that involve male adolescents and youth. Since the number of girls who train in martial arts is increasing, further studies on the effect of martial arts training on the aggression and anger of teenage girl athletes are essential. Therefore, this study was conducted to assess the anger level of teenage girl athletes studying two different forms of martial arts and to compare their scores to those of swimmers and nonathlete teenagers.

## 2. Methods and Subjects

### 2.1. Participants

This case control study was conducted on 291, 11- to 19-year-old adolescent athletes in karate and judo, in Tehran, Iran in 2008. The report of boy athletes was accepted for publication in previous report [13], and in this paper, we report the finding of our study in girl athletes. The inclusion criteria included being a member of a youth sports club and engaging in regular, weekly practice of judo or karate. The studied clubs were selected randomly through cluster sampling from all registered sport clubs of Tehran city. Then, all members of each selected club were entered into the study by census method. In case of lack of individual consent to participate in the study or incomplete filling of the questionnaire (more than 25% of the questions unanswered), the subject was excluded from the study. 

Adolescent swimmer athletes and nonathlete teenagers of public schools in the mentioned age ranges were considered as the control group.

### 2.2. Measures

The “Adolescent Anger Rating Scale” (AARS) questionnaire was designed by DM Burney to measure distinct types of anger [14]. In the AARS, there are subscales: instrumental anger (a negative emotion that triggers a delayed response resulting in a desired and planned goal of revenge and/or retaliation), reactive anger (an immediate angry response to a perceived negative, threatening, or fear-provoking event), and anger control (a method used to respond to reactive and/or instrumental stimulations). Instrumental anger scores range from 20 to 80, and reactive anger scores range from 8 to 32. Higher instrumental or reactive anger scores reflect greater endorsements of instrumental and reactive anger, respectively. Anger control scores range from 13 to 52, and higher scores reflect greater endorsements of anger control [14, 15]. The validity and reliability of the AARS tool has been reported by Burney and Kromrey [15].

After a general explanation to the adolescent and her parent, the questionnaire was completed by the adolescent herself. Demographic data of participants (age, number of brothers and sisters, number of friends, times expelled from school, friends' behavior, and playing another sport) was obtained at the end of the questionnaire. In the athlete group, the questions asked included how many years the sport had been practiced, hours of training per week, and whether the sport was practiced year round or during particular seasons.

The Research Ethics Committee of Tehran University of Medical Sciences approved the study.

### 2.3. Analysis

SPSS software (version 13) was used for data analysis. Quantitative variables including age, number of brothers and sisters, and the score of each anger subscale were presented according to their means (standard deviation, SD). For comparison of quantitative variables, including anger score, one-way analysis of variance (ANOVA) was used, while for categorical variables the chi-squared or the Fisher exact tests were used. Multiple regression analysis was used to determine the probability of any confounding effect of the number of years of participation in the sport, the number of hours of training a week, and age. For adjustment of multiple comparisons, Bonferroni test was employed. *P* values of less than 0.05 were considered statistically significant.

## 3. Results

From the total of 294 questionnaires collected, three individuals were excluded due to the incompleteness of their responses to the questionnaire. Therefore, 291 questionnaires were analyzed. Mean (±SD) age of all participants was 15.49 (±1.93) years. Mean age of swimmers was less than that of other groups. The average (±SD) number of brothers and sisters of the participants was 2 (±1.75). Demographic data of the participants and their sportive past records are shown in Table 1.

Although the number of dropouts from school was higher among the judoka than among other trainers, the difference was not statistically significant (*P* = 0.4). 

The number of years of participation in the sport and the number of hours of training per week did not have a confounding effect on the results of the study. However, age was identified as a confounding variable. In order to make the anger scores of adolescents with different ages comparable, raw scores for total anger scale and all anger subscale were converted to T scores by tables available in the Adolescent Anger Rating Scale Professional Manual [14]. 

Mean (±SD) total anger rate was highest among children who participated in judo, 50.64 (±8.33), and was lowest among swimmers, 44.49 (±6.96). This rate was significantly different between the different groups; *P* < 0.001.  Table 2 shows average T score of adolescents' anger scale and subscales in participants of different groups. As shown, instrumental and reactive anger subscales were also significantly different between groups, while anger control scores did not differ in a statistically significant way.

With regards to total anger scale, the difference was significant between judokas and swimmers (*P* < 0.001) and also between nonathletes and swimmers (*P* < 0.001). In the instrumental anger subscale, the difference was significant only between nonathletes and swimmers (*P* = 0.049). In the reactive anger subscale, the difference was significant only between nonathletes and swimmers (*P* = 0.01). In the anger control subscale, the difference was significant between judoka and swimmers (*P* = 0.008) and also between nonathletes and swimmers (*P* = 0.022). The effect sizes are shown in Table 3.

## 4. Discussion

The purpose of this study was to assess the anger rate among teenage girl martial arts athletes and to compare it to that of nonathletes and athletes of other sports. The results obtained showed that anger rates were higher among judoka and lower among swimmers in comparison to the other groups.

Although aggression in sport is mostly instrumental, this does not make it acceptable for sportsmen to be aggressive [6, 7]. A number of explanations for aggression among athletes have been identified such as prior aggression or provocation from the opponent. Athletes can also be highly ego-oriented, may have a low level of moral development, or may want to show off how tough they are. They may also see it as a part of their role or may feel group pressure to be aggressive. In addition, the more frequently teams compete with each other, the more likely they are to be aggressive in response to peer pressure [7, 16].

### 4.1. Anger and Judo

The results of our study showed that the rate of anger among judoka was higher than among other groups. In the Reynes and Lorant study [3], anger scores in 8-year-old boys who, were beginning judo training, were higher than among their peers. The study proposed the possibility that there may be a difference in aggressiveness between the various martial arts. In their study, there was no significant difference between girls and boys [3]. In another study by these same researchers, carried out after one year of judo training, it was shown that judoka had significantly higher scores in total aggression, verbal aggression, and anger than the control group, indicating that differences were not based on differences among initial anger scores [9]. After a 2-year follow-up, they reported that judo training seemed to have a negative effect on anger scores [10].

In the present study, although total anger scores among judoka were higher than among other groups, the difference was significant only in comparison to the swimmers. When comparing subscale scores, judoka received significantly lower scores of anger control than swimmers, while their instrumental anger scores were significantly higher. Aggression among students is constantly found to be one of the most common reasons for suspension [17, 18]. On the other hand, being suspended or expelled from school is a risk factor for violence and aggression [19]. In the present study, adolescents who trained in judo reported higher rates of suspension from school. However, comparison to other participants in the study yielded nonsignificant differences.

### 4.2. Anger and Karate

In our study, no significant difference in anger scale scores and subscale scores was found between karateka and other groups of athletes and nonathletes. Murray et al. [12], studying aggression in 661 students who participated in a broad range of physical activities, reported that aggression scores were significantly higher among students who participated in aggressive sports, including karate, than among those who engaged in less aggressive ones. But the findings of our study in this field are in agreement with the observations of the Reynes and Lorant. They reported the absence of a significant difference in aggression scores of 8-year-old boys at the beginning of karate training when compared to their peers [3]. In their followup, after one year of traditional judo training, it was shown that judoka became more aggressive while after one year of karate training no significant change of aggressiveness was found [9, 11]. After two years of martial arts training, Reynes and Lorant reported that karate had no effect on aggressiveness while judo had a negative effect on anger scores [10]. The authors suggested that the significant difference in anger scores after 2 years of training in karate or judo could be due to different training programs. For instance, they pointed out the increased prominence of kata and meditation in karate with respect to judo and where kata and meditation were either absent or downplayed leading to increased difficulty in acquiring self-control [10]. However, it does not seem that kata and meditation were associated with measured physical aggression scores [10]. A significant difference of anger scores was not found between judo and karate groups in our study.

### 4.3. The Effect of Age on Athlete Adolescent Aggression Rate

In our study, the mean age of swimmers was less than that of other groups. This is due to the fact that swimming is perceived to be more appropriate for younger children than martial arts, which are viewed by parents as for older children. As a result, and due to the possibility of indirect effects of psychological characteristics of puberty, age was evaluated as a confounding variable.

Studies carried out to measure aggression in early, mid-, and late adolescence have shown a curvilinear (developmental) pattern. The highest rates of aggression have been associated to mid-adolescence group (14 years olds), while the lowest rates are found among the older adolescents group (17 years old) [20, 21]. Possible explanation for increased aggression in midadolescence was included the endocrinologic changes of puberty as well as cognitive processes such as inadequate development of problem-solving skills. Studies have shown a weak relationship between aggression and estrogen in female adolescents or between aggression and testosterone or its binding globulin in males [20, 22]. In this study the mean age of swimmers was also compatible with the peak age of aggression (midadolescence). Hence, a higher aggression rate was expected in this group. On the other hand, with regard to the average age of judoka, which was closer to late adolescence, lower aggression rate was expected. However, total and instrumental anger rates did not decrease along with age increase among judoka, but in fact the anger rates increased, presumably in relation to the type of sport. In other words, the findings of our study are consistent with the possibility that judo training increases anger rate while swimming decreas it. 

If we consider the limited prior studies that exist on martial arts and aggression adolescents, one of the strong points of our study is the proper sample size, allowing us to compare different sports. In contrast, one of the weak points was the impossibility to reach a “definite” conclusion. Cohort studies in this field are recommended. A further limitation was the subjective nature of our data collection, which relied on a self-report questionnaire.

The results of this study found that the anger scores of girls who engaged in judo were higher than among other groups and was statistically significant in comparison to the groups of swimmers. In order to factor in the possibility that psychological traits associated with puberty could indirectly affect the results, anger scores were adjusted by age according to the T-score tables provided by the questionnaire manual [14]. When comparing anger subscales between the different groups, both instrumental and reactive anger scores turned out to vary significantly. In the instrumental anger subscale, the mean score of judo practitioners was higher than the mean score of swimmers, while the mean reactive anger was significantly lower in the group of nonathletes.

Evaluation of anger gives us useful information about the motor counterpart of it, that is, aggression. In order to reach definite results about the relationship between different kinds of sports and anger, and to see if a special kind of sport can be prescribed for adolescents who get high anger scores, more research, especially longitudinal is needed. 

The other field of research is examining the impact of playing combat videogames on children and adolescents. It is reported that violent video games or television violence exposure does not predict aggressiveness in youth [23–25]. It seems to be the same about training martial arts in real life.

## Figures and Tables

**Table 1:** Demographic information of participants by type of sport.

Groups	Age (year)	Experience in sport activity (year)	Training a week (hour)
	Mean (SD)	Mean (SD)	Mean (SD)
Judo (*n* = 70)	16.41 (1.50)	3.59 (2.11)	7.83 (4.89)
Karate (*n* = 66)	14.94 (1.88)	5.39 (2.36)	6.20 (3.46)
Swimming (*n* = 59)	13.73 (2.22)	5.02 (2.42)	5.68 (3.07)
Nonathlete (*n* = 96)	16.28 (0.95)	—	—

SD: standard deviation.

**Table 2:** Total anger scale and anger subscales scores by type of sport.

Groups	Instrumental anger	Reactive anger	Anger control	Total anger
	Mean (SD)	Mean (SD)	Mean (SD)	Mean (SD)
Judo	52.13 (11.74)	47.81 (7.49)	49.94 (8.19)	50.64 (8.33)
Nonathlete	50.89 (9.56)	49.94 (7.96)	50.61 (7.59)	50.15 (8.18)
Karate	49.08 (6.81)	46.73 (6.99)	51.85 (7.78)	47.83 (7.50)
Swimming	46.95 (5.46)	45.97 (7.68)	54.39 (7.58)	44.49 (6.96)
*P* value	0.007	0.007	0.007	<0.001

SD: standard deviation.

**Table 3:** Effect sizes of anger scale and subscale comparison by type of sport.

Sport	Instrumental anger Mean difference	Reactive anger Mean difference	Anger control Mean difference	Total anger Mean difference
	[95% confidence interval]	[95% confidence interval]	[95% confidence interval]	[95% confidence interval]
Karate/Judo	−3.053 [−7.127, 1.021]	−1.087 [−4.541, 2.367]	1,906 [−1.640, 5.451]	−2.810 [−6.380, 0.761]
Karate/Swimming	2.127 [−2.128, 6.381]	0.761 [−2.845, 4.368]	−2.541 [−6.243, 1.161]	3.342 [−0.386, 7.070]
Karate/Non athlete	−1.810 [−5.607, 1.987]	−3.210 [−6.429, 0.009]	1.234 [−2.070, 4,538]	−2.313 [−5.640, 1.015]
Judo/Swimming	5.179 [0.983, 9.376]	1.848 [−1.709, 5.406]	−4.447 [−8.099, −0.795]	6.151 [2.474, 9.829]
Judo/Non athlete	1.243 [−2.498, 4.975]	−2.123 [−5.287, 1.041]	0.672 [−3.919, 2.576]	0.497 [−2.774, 3.768]
Swimming/Non athlete	−3.936 [−7.864, −0.008]	−3.97 [−7.301, −0.641]	3.775 [0.357, 7,193]	−5.654 [−9.097, −2.212]
